# A randomised waitlist-controlled trial protocol for examining the efficacy of a nature-based intervention (angling) for military veterans and emergency service personnel with post-traumatic stress disorder (PTSD)

**DOI:** 10.1186/s13063-026-09506-9

**Published:** 2026-02-13

**Authors:** Nicholas R. Cooper, Mark Wheeler, Guyan Sloane, Claire L. Wicks, Mike Rogerson, Richard Philpot, Sheina Orbell

**Affiliations:** 1 Department of Psychology, University of Essex, Colchester, UK; 2 School of Sport, Rehabilitation and Exercise Sciences, University of Essex, Colchester, UK; 3 Essex ESNEFT, Psychological Research Unit for Behaviour, Health and Wellbeing, Colchester, UK

**Keywords:** PTSD, Nature-based social prescribing, Nature-based interventions, Veterans, Depression, Anxiety, Wellbeing, Angling, Fishing, Peer support, Green exercise, Ecotherapy, Veteran, Police, Fire service, Ambulance, Coast guard, ESP, NBI, Emergency service personnel

## Abstract

**Background:**

Post-traumatic stress disorder (PTSD) is a severely debilitating psychological condition that can affect one’s capacity to work and well-being. Nature-based interventions (NBI) offer a unique avenue for treatment without the typical barriers current psychotherapeutic treatments create for veterans and emergency service personnel (ESP). Data from a pilot study involving military veterans suggests angling as one such NBI, but large-scale robust experimental work is required to assess the causal impact of NBIs. We will therefore conduct a full-scale randomised controlled trial powered to provide definitive evidence of the efficacy of a nature-based, group angling intervention for military veterans and emergency service personnel with PTSD.

**Methods:**

We will recruit 264 participants for a randomised waitlist-controlled trial of a 2-day, 1-night nature-based angling intervention. We will compare PTSD, anxiety, depression, and wellbeing outcomes between an active and control group. The primary outcome relates to the comparison of pre-intervention (2 weeks before the intervention), with post-intervention measures for the active and control groups taken at 2 and 4 weeks after the intervention. We will also assess if the changes in PTSD scores in the active group might be considered clinically significant and reliable by comparing baseline (2-week pre-intervention) and 2-week and 4-week post-intervention PTSD scores. Secondary outcomes include examining loneliness, expressed anger using baseline and 4-week post-intervention scores. Long-term efficacy will be assessed by 6- and 12-month follow-up surveys. Cost-effectiveness will also be assessed. Process analyses will include examining the contribution of mood change, nature restoration, peer support, and skill learning experiences to the outcomes during the intervention scores at the beginning and end of the intervention.

**Discussion:**

This randomised controlled trial (RCT) will evaluate the efficacy of a nature-based intervention in reducing PTSD symptoms in military veterans and frontline workers such as emergency service personnel and contribute to the evidence base on the clinical impact of NBIs for mental health outcomes.

**Trial registration:**

ISRCTN13593648. Registered on 19 June 2024

## Administrative information

Note: The numbers in curly brackets in this protocol refer to SPIRIT checklist item numbers. The order of the items has been modified to group similar items (see http://www.equator-network.org/reporting-guidelines/spirit-2013-statement-defining-standard-protocol-items-for-clinical-trials/).

**Table:** 

Title {1}	A randomised waitlist-controlled trial protocol for examining the efficacy of a nature-based intervention (angling) for military veterans and emergency service personnel with post-traumatic stress disorder (PTSD)
Trial registration {2a and 2b}	Name of the registry: ISRCTN Trial registration number: ISRCTN13593648 Date of registration: 19 June 2024 URL of trial registry record: 10.1186/ISRCTN13593648
Protocol version {3}	Version 1; 01/03/2024
Funding {4}	This project is funded by the National Institute for Health and Care Research (NIHR) [Policy Research Programme (PRP) Round 30 Nature-Based Social Prescribing (NIHR206791)]. The views expressed are those of the author(s) and not necessarily those of the NIHR or the Department of Health and Social Care
Author details {5a}	^1^Department of Psychology, University of Essex ^2^School of Sport, Rehabilitation and Exercise Sciences, University of Essex ^3^Essex ESNEFT (East Suffolk and North Essex NHS Foundation Trust) Psychological Research Unit for Behaviour, Health and Wellbeing (EEPRU) ^*^Corresponding author email: ncooper@essex.ac.uk
Name and contact information for the trial sponsor {5b}	Dr. Mantalena Sotiriadou, REO, University of Essex, CO43SQ ms21994@essex.ac.uk
Role of sponsor {5c}	The sponsor will play no part in study design; collection, management, subsequent analysis and interpretation of data; writing of the report; and the decision to submit the report for publication.

## Introduction

### Background and rationale {6a}

This project addresses the critical issue of post-traumatic stress disorder (PTSD) in military veterans and emergency service personnel. PTSD, characterised by trauma-induced symptoms, often results in impaired functioning and comorbid conditions such as depression and anxiety [1, 2]. Existing psychotherapeutic treatments are associated with significant barriers for veterans and others, including delayed help-seeking, lengthy waiting times, and a lack of military-specific knowledge among healthcare professionals [3]. In response to these challenges, this study proposes a nature-based angling intervention as a novel approach, leveraging the potential benefits of nature-based interventions (NBIs) for mental health.

The need for innovative treatments is underscored by the limitations and reduced effectiveness of current therapies for veterans [4, 5]. The proposed angling intervention, developed over 7 years, aims to provide a local, cost-efficient, and accessible solution. Previous trials indicated significant improvements in PTSD, anxiety, and depression following a single 2-day intervention, with peer-supported angling proving particularly effective [6]. This study’s inclusion of emergency service personnel acknowledges their elevated risk of PTSD and seeks to provide much-needed evidence for NBIs in this understudied population.

The choice of angling as the intervention is justified by its broad appeal, accessibility, and potential for year-round engagement. The intervention’s delivery is facilitated by a not-for-profit community interest company (iCARP CIC), emphasising collaboration with military veterans and aligning with broader initiatives that promote angling for well-being. The research commissioned by the NIHR-PRP aims to contribute robust experimental evidence of the causal impact of NBIs to support the integration of NBIs into mental health care policy, potentially reducing the social and economic costs of untreated PTSD within the community.

We aim to conduct a fully powered randomised waitlist-controlled trial of an angling based NBI for people with PTSD. This study builds upon a developmental phase of the project, where the feasibility of the intervention and all measures were rigorously tested and validated. It represents a significant step towards addressing the mental health needs of veterans and emergency service personnel, offering a promising alternative to conventional treatments. The outcomes may not only improve the well-being of these populations but also inform broader mental health policies, emphasising the role of nature-based interventions in promoting mental health and resilience.

### Objectives {7}

- Primary outcome objectives
  - Assess mean changes in mental health outcome measures (e.g. PTSD, see item “Outcomes {12}” for a full list) from pre- to post-intervention between active and control groups (see item “Statistical methods for primary and secondary outcomes {20a}” for analysis details).
- Secondary outcome objectives
  - Assess proportion of participants achieving clinically significant change and reliable change between 2-week pre- and 4-week post-intervention PTSD scores.
  - Conduct sub-group analyses to evaluate mean change in primary outcomes by stratification factors (military veteran vs. ESP, PTSD severity, gender).
  - Assess mean change in social loneliness, work and social adjustment, and anger expression from pre- to post-intervention between active and control groups.
  - Assess mean change in primary outcome measures from baseline to 6 months and 12 months for the active intervention group only.
  - Assess mean change in process measures (e.g. mood, see item “Outcomes {12}” for a full list) and physiological data (heart rate variability and eye movement) from day 1 to day 2 of the NBI experience.
  - Assess the relationship between intervention experiences and changes in process measures from day 1 to day 2 of the intervention and their relationship to change in outcomes.
  - We will also explore the cost-effectiveness and social return of investment of the intervention.

### Trial design {8}

A randomised waitlist-controlled superiority trial with an allocation ratio of 1:1 and a 4-week follow-up. The primary outcome will be assessed at 2-week and 4-week post-intervention and compared with the waitlist control group. The total follow-up period is 12 months (intervention group only).

## Methods: participants, interventions, and outcomes

### Study setting {9}

The intervention will be run by iCARP CIC at their “Lifted Lakes”, a single mental health and wellbeing centre in Great Oakley, Essex, UK.

### Eligibility criteria {10}

This includes UK armed forces veterans or emergency service personnel (current or former). Therefore, those not a military veteran or member of the emergency services (current or former) will be ineligible. Angling coaches, delivering the intervention, require an Angling Trust Level 2 Coaching Certificate.

### Who will take informed consent? {26a}

The registration form, delivered via an online platform (Qualtrics), provides further information for participants and a mechanism for providing informed consent.

### Additional consent provisions for collection and use of participant data and biological specimens {26b}

The consent form asks if participants would be happy to be contacted in the future about research participation. Participants can say no to this and still part in the study.

## Interventions

### Explanation for the choice of comparators {6b}

A randomised, waitlist control group will be used as the comparator. With an intervention such as this, it is not possible to employ a well-balanced placebo condition, and a waitlist control is therefore commonly used in psychological studies. It is also an ethical alternative to a no-treatment option, as all participants receive the intervention eventually.

### Intervention description {11a}

The intervention is named *Casting Away Trauma* (CAT). Following our preliminary work and the developmental phase of the current project, we have a well-established and manualised protocol for the intervention:

- Day 1 am — Arrival at fishing lake, introduced to personal angling coach, shown to designated lakeside “swim” with tent and seat, health and safety briefing, set-up equipment, and recording of “arrival” process measures
- Day 1 pm — Fishing instruction, fishing and social interaction, evening meal, and participants fish through the night or sleep as they choose
- Day 2 am — Breakfast, fishing, and social interaction
- Day 2 pm — Fishing and pack-up equipment, recording of “departure” process measures, instructions on how to keep in contact via social media, etc.

Prior to their intervention, participants will be sent a letter and an infographic developed for the study visualising what to expect at the intervention. The venue will be available exclusively to participants. Professional angling coaches with Angling Trust certification will be provided at a ratio of 1:2 participants. As recommended by Husk et al. [7], participants will be supported to travel to the venue. iCARP’s “Lifted Lakes” venue has a covered socialising area with a fire pit, and participants are encouraged to bring warm clothing, since socialising with peers is a key component of the intervention. Participants are free to move around the lake and talk to other participants and use the communal area for socialising and taking warm drink breaks. Food will be cooked and shared by iCARP volunteers. At the end of the experience, participants will be encouraged to join a “Facebook” group in order to keep in contact via social media and will be offered a selection of exit routes. During this period, the wait group receives no intervention and continue with their normal day-to-day activities and usually care.

### Criteria for discontinuing or modifying allocated interventions {11b}

Participants may drop out of the study without giving a reason. However, any data collected up to the point of withdrawal will be retained unless requested otherwise. Criteria for discontinuation during the 2-day intervention period is handled by a mental health specialist on a 1 to 1 basis. Should mental health first aid be required during the intervention, the participant will be given the option to discontinue or continue with the intervention, and a collaborative decision can be formed as to the future support required, and then referrals to appropriate external services can be made if required.

### Strategies to improve adherence to interventions {11c}

The intervention takes place over 2 days/1 night. Attendance logs are taken of active group participants arriving for the intervention. In the week prior to the intervention, participants are contacted by telephone and reminded how to find the venue. They are also given reassurance about what they will have to do during the 2-day intervention. Participants who attend the intervention are also compensated £50 GBP towards their travel expenses. Waitlist control group activities are not monitored.

### Relevant concomitant care permitted or prohibited during the trial {11d}

Usual care for participants continues throughout the trial. There is nothing prohibited.

### Provisions for posttrial care {30}

As this trial consists of one (per participants) nondrug intervention that lasts 2 days/1 night, there is minimal risk other than risks associated with being near water. In case of psychological distress (e.g. “flashbacks”), a mental health professional will be present at all interventions in order to observe and monitor for any signs of distress. The mental health professional’s role will be to administer mental health “first aid” to any participants whose PTSD symptoms might be triggered during the day. First aid will include grounding techniques, emotional regulation procedures, and psycho education around the symptoms they may be experiencing. Signposting will also be given, where access to additional care can be sought. In the week prior to the intervention itself, the mental health professional contacts participants attending to help alleviate any apprehension participants might be feeling about attending the intervention.

Following consultation with various mental health and wellbeing service care providers, we appreciate the importance of having a targeted and varied exit strategy for participants leaving the study. Consequently, we have devised an array of options for our participants allowing a choice of levels of engagement:

- Do not wish to fish again.
- Wish to fish again independently
- Wish to fish again with advice from iCARP CIC
- Wish to fish again with iCARP CIC
- Wish to volunteer to help iCARP CIC
- Wish to train to be an iCARP CIC angling coach

Participants will indicate which of these five options they prefer at the 4-week follow-up. At the end of the 2-day intervention, participants will be given a “Keep in Touch” information sheet that will include these options and an invitation to 

#### Compensation for harm

The University of Essex provides indemnity against negligent harm caused as a direct result of an employee’s or a student’s actions. The chief investigator is an employee of the University of Essex.

### Outcomes {12}

#### Primary outcome measures

- The following measures will be employed to evaluate the effectiveness of the intervention and will be assessed 2 weeks before the intervention and 2-week and 4-week post-intervention. The National Institute for Health and Care Excellence (NICE) [8], recommends the use of self-report measures for evaluation of outcome in PTSD interventions. These measures of PTSD, anxiety, depression, and wellbeing were used in the development phase, establishing their acceptability and sensitivity to change over the short to medium term: PTSD symptoms are assessed by the PTSD Checklist for DSM-5 (PCL-5) [9], Depression by the Patient Health Questionnaire-9 (PHQ-9) [10], and Anxiety by the General Anxiety Disorder-7 Scale (GAD-7) [11]. Participants will also complete the 7-item Short Warwick-Edinburgh Mental Wellbeing Scale (WEMWBS) [12] to evaluate positive feelings of wellbeing. Mean change from baseline will be assessed for each of these outcomes for active intervention and control groups.

#### Secondary outcome measures

Use subheadings (or just numbers that correspond to the objectives) under this heading following the same order as in the introduction statement of objectives:

- Using scores on the PCL-5 [9], we will assess the proportion of participants achieving clinically significant change and reliable change between 2-week pre- and 4-week post-intervention in the following ways: clinically significant change equates to a decrease in PTSD score of greater than or equal to 10, and reliable change reflects a decrease in PTSD score of greater than or equal to 5.
- Sub-group analyses will be carried out to evaluate mean change in the primary outcome measures (described above) by stratification factors (military veterans vs emergency service personnel), PTSD severity (high vs low), and gender (male vs female).
- The following measures of loneliness and expressed anger were included in the developmental phase and will be assessed as follows: Loneliness will be assessed by the Short Version of the Social and Emotional Loneliness Scale for Adults (SELSA-S) [13]: 2 weeks prior and 2- and 4-week post-intervention. Expressed anger will be assessed by the State–Trait Anger Expression Inventory (STAXI) [14], which measures how frequently participants have expressed anger verbally or physically in the last month. This will be taken 2-week pre-intervention and 4-week post-intervention. The Work and Social Adjustment Scale (WSAS) [15] is a 5-item measure of impairment in general social functioning. This will be measured 2-week pre-intervention and at a 6-month and 12-month follow-up since it will not be sensitive to short-term change. Mean change from baseline will be assessed for each of these outcomes for active intervention and control groups.
- Follow-up measures at 6 and 12 months: It is not possible to include long-term follow-up measures in the RCT because the waitlist group will receive the intervention after the RCT 4-week data collection is completed. Instead, we will take measures of PTSD symptoms, anxiety, depression, wellbeing, loneliness, expressed anger, and social functioning, to provide pre- and post-data from the intervention fishing group only at 6 months and 12 months after their intervention. We will also measure changes in NHS (National Health Service) service use and employment at these times. In addition, to gauge the impact of the NBI on long-term behaviour and engagement with nature-based activities, we will follow-up on the participant exit strategy to assess how participants have engaged with angling or other nature-based activities since the intervention. Mean change from baseline to follow-up will be assessed.
- Mean changes in process measures (mood and physiological changes) will be assessed between the beginning of day 1 of the intervention to the end of day 2. We will evaluate mood using the brief Profile of Mood States (POMS) [16]. Our developmental phase has shown that these measures are sensitive to detect change between arrival and departure at the intervention. Following consultation in the development phase, we include objective physiological measures of psychological states: pulse heart rate and heart rate variability and assess hypervigilance using a mobile eye-tracking device to quantify saccadic behaviour.
- *Measures of the hypothesised active elements of the intervention*: We developed an item pool during the developmental phase. These measures will be taken prior to departure. Eight items from the Perceived Restorativeness Scale (PRS) [17] measure perceived experience of restoration from the environmental surroundings. Bespoke questions have been developed to evaluate self-efficacy, experience of fishing, and being in a group of veterans. Fourteen questions explore participants’ experience of fishing (e.g. “I found I could focus on the fishing”). Eleven questions explored participants’ experience of peer support over the weekend (e.g. “I felt the people around me understood”). These questions are answered by participants at the end of day 2 of the intervention. We will assess the relationship between intervention experiences and changes in process measures and any potential interaction with outcome measures over the course of the trial.
- The cost-effectiveness and social return of investment will be analysed based on the Greater Manchester Cost-Benefit Analysis Model [18]. This will allow for comparisons to be made with other treatments and studies using government-approved values.

### Participant timeline {13}

**Fig. 1:**
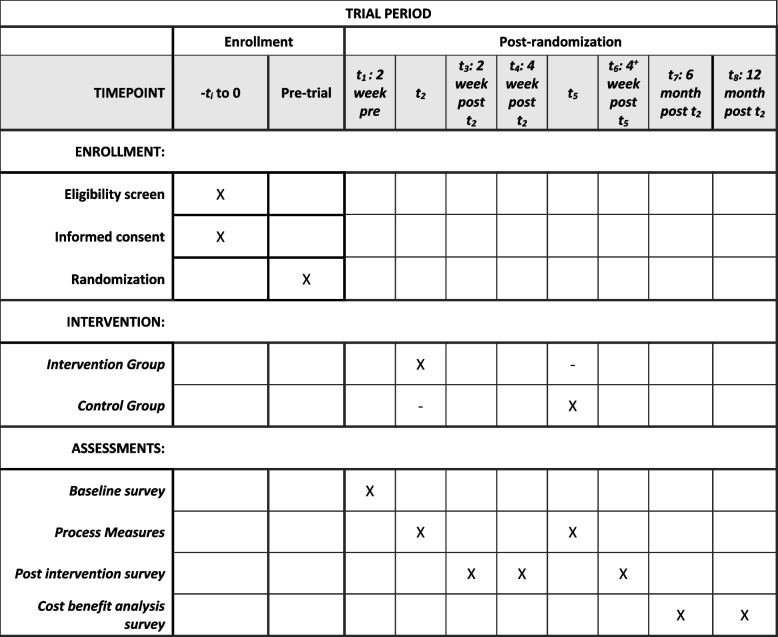
SPIRIT timeline diagram

**Fig. 2:**
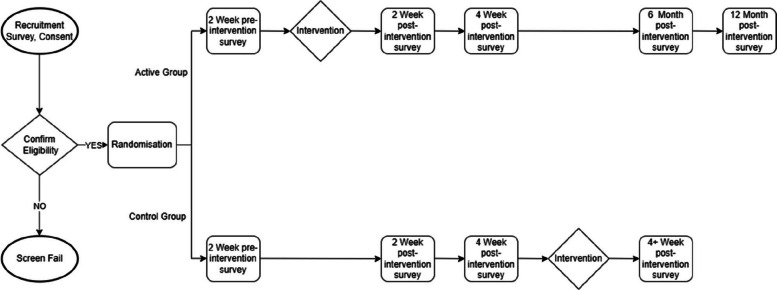
Casting Away Trauma participant pathway

The study protocol follows the SPIRIT 2025 statement recommendations (Fig. 1). See Fig. 2 for the pathway for each arm.

### Sample size {14}

#### Power analysis

Our main dependent variable will be PTSD symptoms (as measured using the PCL-5). The results from our developmental phase showed a significant group × time interaction for PCL-5 scores, with significant improvements at 2-week and 4-week post-intervention for the angling but not the control group. Given that the purpose of the NIHR call is to build robust evidence for the clinical impact of NBIs, it is imperative that study power should be based on the main DV of interest with the lowest difference scores (i.e. a difference score that will allow the power calculation of a sample large enough to detect a significant effect (should there be one). This is the effect size observed between groups at 4-week post-intervention in the developmental phase (Cohen’s d = −0.52). For a 2-tailed test with 95% power, an alpha of 0.05, and *d* = −0.52 effect size, the power analysis provides a sample size of *n* = 196 (98 participants in each group).

#### Recruitment sample size

The attrition rate after randomisation in the development phase was approximately 25%. We aim to randomise our power analysis sample size + 30% for the main phase. Thus, we aim to randomise 196 + 30% = 254.8 participants (rounded to 264: 132 intervention, 132 controls to facilitate 12 participants per intervention). By rounding to 264, we can ensure slots are filled across 22 scheduled sessions of 12 participants each. We have designed an RCT with 11 active intervention groups and 11 waitlist control interventions to be delivered after RCT data are collected. We will run eight angling interventions (four active and four controls) sequentially (a maximum of *n* = 12 participants in each session) in years 1 and 2 and six interventions (three active and three controls) in year 3.

### Recruitment {15}

Recruitment into the study will be via on online Qualtrics link embedded in the project website and on all recruitment materials. Potential participants can either engage via the project’s webpage or contact the study team via email or telephone. Participants complete a registration form via a Qualtrics platform in which demographic and PTSD severity (via PCL-5 items) information are requested via questionnaire items for purposes of randomisation. Alternative arrangements, such as paper copies of questionnaires, will be provided where requested by participants to ensure inclusivity; participants will be made aware of such options within recruitment materials and communications. Anonymity will be ensured whatever the media used. The website link permits self-referral, enabling us to reach those suffering from PTSD who are not receiving support from either NHS or charitable organisations.

#### Routes to promote direct self-registration

The development phase showed that the majority of participants were recruited via social media platforms including Facebook. We will advertise the study via the social media platforms (Facebook, Twitter, and Instagram) of organisations currently sponsoring iCARP work (angling companies Korda and Nash, which combined have over 527,000 followers on Facebook and 504,000 on Instagram). We will employ three Facebook “pushes” as we did in the development phase to specifically target military veterans and ESPs with particular attention to targeting minorities and women.

#### Routes to promote self-registration via referring agencies

We will provide stakeholders with capacity to refer/inform potential participants to access the online registration with a flier and information pamphlet that describes what the intervention has to offer as an extension to their existing provision. Feedback will be sought on draughts of the pamphlet from stakeholders, service providers, and our PPI. All recruitment materials are designed to convey inclusivity across gender, ethnicity, and religion via considered inclusion of diversity in imagery and messaging.

Stakeholders have been identified broadly as charities representing veterans and ESPs, Occupational Health Departments of ESPs, ESP outreach events and organisations, and social prescribers.

## Assignment of interventions: allocation

### Sequence generation {16a}

Participants will be allocated to the control or waitlist condition by covariate adaptive randomisation, a valid technique for clinical trials [19, 20] where specific participant characteristics (covariates) can be controlled for on a sequential basis based on recruitment and allocation to date [21, 22]. Randomisation will be implemented using Sealed Envelope (https://www.sealedenvelope.com), an online randomisation service. It applies a minimisation algorithm with a random component. The minimisation algorithm will balance groups on the following pre-specified covariates: gender, PTSD symptom severity (PCL-5 score) [9], and service group (military veterans vs nonmilitary service personnel vs a mix of both). PTSD symptom severity will be dichotomised into low and high categories using a fixed median split based on baseline PCL-5 scores from the initial cohort of participants enrolled prior to commencement of full randomisation (*N* = 48). This median cutoff-point will be calculated once and applied consistently to all subsequently enrolled participants and will not be updated during recruitment. For each new participant, the algorithm will calculate the marginal imbalance that would result from assignment to each group across the stratification variables. Participants will be assigned to the group that minimises overall imbalance, with a probabilistic random element (e.g. 80% allocation to the group minimising imbalance) to maintain allocation concealment and reduce predictability. The randomisation schedule will be devised prior to recruitment using an online tool and applied to eligible participants recruited online after participant identification numbers have been assigned.

### Concealment mechanism {16b}

Allocation concealment will be ensured using an automated, web-based randomisation system (Sealed Envelope, https://www.sealedenvelope.com), which implements the minimisation algorithm centrally. The randomisation sequence will be generated and applied in real time by the system and will not be accessible to the researchers involved in participant recruitment or enrolment.

Group assignment will only be revealed by the system after participants have been deemed eligible, provided informed consent, and been assigned a participant identification number. This procedure prevents the enrolling party from predicting or influencing the upcoming allocation.

### Implementation {16c}

For stratification purposes, participants are randomised after recruitment surveys are completed using covariate adaptive randomisation, which is completed by the senior research officer (GS). The senior research officer is also the person who confirms final eligibility and processes the registration to enrol each participant.

## Assignment of interventions: blinding

### Who will be blinded {17a}

Due to the nature of the intervention, trial participants and those administering the intervention (e.g. angling coaches) are blinded during the intervention. As all outcome measures are anonymously self-reported, those involved in data analyses will be blind to group allocation.

Participants are not informed about which group (active vs control) they are placed in, nor are they informed of the differing survey timings between groups. Both groups eventually attend the intervention, which helps blind participants as both groups complete surveys before and after the intervention. Separate pre- and post-surveys were created and administered to each group in order to specifically tailor the wording for each group and keep them blinded. Angling coaches are blinded as they do not know if a particular participant is in the active intervention group or attending the intervention as a waitlist control group participant. All participants (eventually) take part in the angling weekend and are all treated exactly the same by the angling coaches.

### Procedure for unblinding if needed {17b}

There is no requirement for emergency unblinding procedures because the trial is waitlist controlled, and all participants eventually attend the 2-day behavioural intervention.

## Data collection and management

### Plans for assessment and collection of outcomes {18a}

All (unless otherwise requested) pre- and post-intervention data will be collected via the online platform Qualtrics. Upon request, data collected via post will be anonymously returned and manually input into a Qualtrics data file. During the intervention, participants, through the use of ID numbers and blank envelopes, anonymously self-report using pen and paper. At recruitment, participant characteristics will be collected and recorded: socio-demographics (age, gender, heritage), nature of trauma experienced, duration of PTSD, previous or current treatment/psychotropic medication prescription and use, living circumstances (alone, with a partner/friend/housemate), romantic relationship status, children, current and past employment status, income, and health service use. Postcodes will be collected for transformation into an established small area deprivation index such as the Carstairs [23]. See the “Participant timeline” section for data collection timings.

### Plans to promote participant retention and complete follow-up {18b}

All participants who fail to complete a survey are sent an email reminder 4 days after initially receiving the survey. If not completed within 2 days of this email reminder, participants are sent a text reminder to their mobile phones.

### Data management {19}

All participants are allocated a random number at recruitment using Qualtrics’s random number generator. All the following data files are password protected and securely stored. The Master File, obtained at recruitment, will be used to identify participants. The data coordinator only accesses it, while all subsequent data files are accessible to all researchers. An anonymous data file with all personal information removed is used for data analysis. A data file with personal details but no outcome data is used for communication and tracking of participants’ progress. Subsequent data files generated from pre- and post-intervention surveys contain no personal information. Paper surveys have no personal information and are stored in a secure room in a locked cabinet before input digitally.

### Confidentiality {27}

All collected information will be kept strictly confidential and will be stored in accordance with the UK Data Protection Act 1998. Personal data is collected at recruitment using Qualtrics (considered secure storage). All files containing personal information are password-protected and shared on Box.com, a password-protected file sharing platform.

### Plans for collection, laboratory evaluation, and storage of biological specimens for genetic or molecular analysis in this trial/future use {33}

N/A. No biological specimens are collected.

## Statistical methods

### Statistical methods for primary and secondary outcomes {20a}

#### Primary outcome analyses

- The key analysis relates to the comparison of pre- (2 weeks before the intervention) and post-intervention measures for the intervention and control groups taken at 2 and 4 weeks after the intervention). A mixed MANOVA with one between-group factor (intervention vs. waitlist control) and repeated measures on all outcomes (baseline vs. 4-week post-intervention) will be conducted.

#### Secondary analyses

- We will assess if the changes in PTSD symptoms observed might be considered clinically significant or reliable. We will assess clinically significant change (a decrease of > = 10 points on the PCL-5) and reliable change (as decrease of > = 5 points on the PCL-5) between the baseline and 2-week and 4-week post-intervention. All analyses will be carried out on veterans and emergency service personnel combined.
- Subgroup analysis of primary outcomes by stratification factors will be conducted using mixed MANOVA (intervention vs waitlist) × repeated measures on all outcomes (baseline vs 4-week post-intervention) separately for each stratification factor.
- A mixed MANOVA with one between-group factor (intervention vs waitlist control) and repeated measures on secondary outcomes (loneliness, expressed anger, work and social adjustment)
- Within-participant analyses will be used to assess change from baseline to 6 months and 12 months for the intervention group only.
- A psychometric analysis of process measures will be conducted via factor analysis and reliability analysis. Path analyses will explore the relationship of process measures regarding the intervention experience to mood change assessed via the POMS questionnaire and psychophysiological data during the intervention and change in outcomes.
- See item e. above.
- A cost-benefit analysis will be used to analyse the cost of effectiveness and social return of investment of intervention based on the Greater Manchester Cost-Benefit Analysis model, which allows for comparisons with other treatment/projects based on government-approved values.

### Interim analyses {21b}

The intervention is brief, consisting of a 2-day/1-night weekend, and follow-up surveys occur within 4 weeks. The trial is subject to a power analysis for a final sample size of 196 with no early stopping and no interim analyses. The intervention is minimal risk due to the nondrug nature of the intervention. Injuries occurring on the intervention are reported, and this does not require an interim analysis.

### Methods for additional analyses (e.g. sub-group analyses) {20b}

In order to check whether the equivalence between the active and control groups was equal before the intervention, we run independent samples *t*-tests or chi-square tests for the service record, health, and sociodemographic characteristics (PTSD score, self-assessed impact of PTSD, physical disabilities, medication, age, ethnicity, relationship status, employment status, annual income, fishing experience, social contact).

### Methods in analysis to handle protocol non-adherence and any statistical methods to handle missing data {20c}

There is no protocol for non-adherence because of the brief nature of the intervention. Analysis will be per-protocol (efficacy) and intention-to-treat (effectiveness). We will evaluate the extent of missing data. We do not plan to impute missing values but may consider multiple imputations or maximum likelihood analyses. A detailed statistical analysis plan will be agreed to before the end of data entry.

### Plans to give access to the full protocol, participant-level data, and statistical code {31c}

Once the project is finished, all data (excluding personal, identifying data) and statistical code will be uploaded to ISRCTN registry, a registry specifically for clinical research.

## Oversight and monitoring

### Composition of the coordinating center and trial steering committee {5d}

The *Core Project Management Team (CPMT)* comprises PI NC and Co-Is S. O. and M. R. who will meet weekly along with the appointed RAs. They will be responsible for the recruitment, delivery of the intervention and its evaluation, analysis, and reporting. The FT RA will hold responsibility for the day-to-day running of the project, the management of the online data collection, and principle analyses, updating the literature review and preparing draft outputs. The PT RA will assist the FT RA with the running of the project and take responsibility for social media comms, angling coaches (including training oversight), and roll-out pilot. Both RAs will collect process measures.

A wider *Project Advisory Group (PAG)* will meet for 10× half-day meetings during the course of the project. The PAG includes the CPMT and all PPI representatives, whose inclusion will ensure their contribution to the recruitment strategies, design and administration of measures, and interpretation of data and dissemination.

A *Main Phase Steering Group (MPSG)* will comprise all members of the PAG plus project collaborators. Six MPSG meetings have been scheduled to ensure proper oversight of the main phase study.

### Composition of the data monitoring committee and its role and reporting structure {21a}

Given the minimal risk of this intervention and its brief duration, an independent Data Monitoring Committee is unnecessary. Participant safety during the intervention will be the responsibility of attending researchers and coaching staff. Any adverse events will be reported to the principal investigator and documented (see item {22}).

### Adverse event reporting and harms {22}

Potential adverse events and harms include the following: slips, trips, and falls, immersion in water, water-borne diseases/infection, thunder and lightning, exposure to animal waste, psychological distress/PTSD flashbacks, etc. Mitigations for such adverse effects include the following: pre-event inspection of lakeside, initial safety talk to participants, observation by coaches, provision of hand-washing facilities and hand sanitising gel, and a mental health professional will attend to observe and monitor any signs of distress. The mental health professional’s role will be to administer mental health “first aid” to any participants whose PTSD symptoms might be triggered during the intervention. First aid will include grounding techniques, emotional regulation procedures, and psychoeducation around the symptoms they may be experiencing.

Serious adverse events (SAE, untoward occurrences that result in harm) are reported by the PI to the sponsor within 24 h of becoming aware of the event. The sponsor reviews all reports within 2 days of receiving the report, and the outcome is recorded in the site management file and the sponsors’ research governance committee. SAEs that are determined to be related and unexpected are reported to the ethics committee.

#### Nonserious adverse events

Written records are made by those running the intervention in the Health Incident Report form and reported to the PI. These forms are reviewed by PI and checked to see if further action is required.

### Frequency and plans for auditing trial conduct {23}

The intervention is brief, low risk, on a single site, and is a behavioural intervention, and as such, there are no plans for auditing trial conduct. The research team are responsible for monitoring all aspects of the ongoing procedures.

### Plans for communicating important protocol amendments to relevant parties (e.g. trial participants and ethical committees) {25}

Changes to the protocol require ethical approval from the sponsors, the internal University Ethics Committee, and the Health Research Authority.

### Dissemination plans {31a}

The primary outcome will be the analysis of change in PTSD symptoms pre- and post-intervention for intervention groups versus waitlist control groups. This will be disseminated in the following ways:

- Liaise with the Department of Health and Social Care (DHSC) to hold a workshop on NBIs for policymakers.
- Finalise intervention manual to ensure consistent intervention delivery going forwards.
- Create plain English information pamphlet to provide clear information about the intervention.
- A written report to the funder
- Papers submitted for publication in leading academic journals
- Presentations at academic/health/environmental conferences and forums
- Dissemination events at the University of Essex and elsewhere to publicise the study findings, to distribute our plain English summary, and to develop further ties with service providers

## Discussion

This study aims to address the critical issue facing military veterans and emergency service personnel, namely the barriers to standard psychotherapeutic provisions by providing evidence for a nature-based alternative. Importantly, this trial will employ a large-scale adequately powered randomised controlled trial sensitive enough to detect clinically significant changes in PTSD symptoms. Beyond PTSD symptoms, this study will rigorously investigate comorbid symptoms of PTSD, potential mechanisms to recovery, and the cost-effectiveness of the intervention. No such large-scale study has previously investigated the efficacy of angling as a nature-based intervention to help reduce PTSD symptoms in military veterans and emergency service personnel.

Some practical issues involve recruitment and retention from target populations that are sometimes difficult to engage in research. For example, one barrier to entry is not recognising that symptoms are psychological [3], so that some people from this particular population will not engage in research addressing psychological illness. This difficulty in engagement also makes ensuring the completion of follow-up surveys difficult. Indeed, pilot data indicates attrition rates after randomisation as high as 25%. Recruitment targets have therefore been adjusted accordingly.

The findings will provide valuable causal evidence on the potential of nature-based interventions to support recovery from PTSD and inform future policy and commissioning of social prescribing initiatives.

### Trial status

The study is currently recruiting. Recruitment began in March 2024 and ends September 2026. Currently, we are attempting to expand the participant pool by including tube/train drivers and serving military personnel. However, that ethical approval process is ongoing. The current protocol is version 1 (01/03/2024).

## Data Availability

The datasets generated during and/or analysed during the current study are available from the corresponding author on reasonable request. Once the project is finished, all data (excluding personal, identifying data) and statistical code will be uploaded to ISRCTN registry.

## Abbreviations

Angling Trust: National Governing Body for Angling in England
CIC: Community interest company
DHSC: Department of Health and Social Care
ESNEFT: East Suffolk and North Essex NHS Foundation Trust
ESP: Emergency service personnel
EEPRU: Essex ESNEFT Psychological Research Unit for Behaviour, Health and Wellbeing
GAD-7: General Anxiety Disorder-7 scale
NHS: National Health Service
NBI: National Health Service
NIHR: National Institute for Health and Care Research
NICE: National Institute for Health and Care Excellence
PHQ-9: Patient Health Questionnaire-9
PRS: Perceived Restorativeness Scale
PRP: Policy Research Programme
PTSD: Post-traumatic stress disorder
POMS: Profile of Mood States
PCL-5: PTSD Checklist for DSM-5
RCT: Randomised controlled trial
SAE: Serious adverse effects
STAXI: State–Trait Anger Expression Inventory
WEMWBS: Warwick-Edinburgh Mental Wellbeing Scale
WSAS: Work and Social Adjustment Scale
SELSA-S: Short Version of the Social and Emotional Loneliness Scale for Adults
